# Cholera resurgence potentially induced by the consequences of climate in the El Niño phenomenon: an urgent call for strengthened cholera elimination in Africa

**DOI:** 10.11604/pamj.2023.46.96.42258

**Published:** 2023-12-04

**Authors:** Nadège Taty Makuntima, Didier Bompangue, Sandra Moore, Nancy Meschinet de Richemond, Thierry Vandevelde, Dieudonné Mwamba, Rita Colwell, Jean Jacques Muyembe

**Affiliations:** 1 Service d’Ecologie et Contrôle des Maladies Infectieuses, Faculté de Médecine, Université de Kinshasa, République démocratique du Congo,; 2 Laboratoire de Géographie et d´Aménagement de l´Espace de Montpellier, Université Paul Valéry Montpellier 3, France,; 3 Programme National d´Elimination du Choléra et de Lutte Contre les Autres Maladies Diarrhéiques, Ministère de la Santé, Hygiène et Prévention, République démocratique du Congo,; 4 Laboratory Chrono-Environnement, UMR 6249, University of Bourgogne Franche-Comté, France,; 5 Veolia Foundation, Aubervilliers, France,; 6 Institut National de Santé Publique, Kinshasa, République démocratique du Congo,; 7 Maryland Pathogen Research Institute, University of Maryland, MD, College Park, United States of America,; 8 University of Maryland Institute for Advanced Computer Studies, University of Maryland, College Park, MD, United States of America,; 9 CosmosID Inc, Rockville, MD, United States of America,; 10 Institut National des Recherches Biomédicales, Kinshasa, République démocratique du Congo

**Keywords:** El Niño, climate, cholera, Africa, cholera elimination, cholera control, *Vibrio cholerae*

## Abstract

A resurgence in cholera cases has been observed throughout Africa during the first half of 2023. Among the many factors that drive cholera transmission, the ongoing climate phenomenon El Niño is likely to continue until March to May 2024. To prevent further cholera spread, it is critical to strengthen cholera control efforts in Africa.

## Commentary

In 2010, the cholera epidemic in Haiti drew international attention to a disease that had been largely ignored [1]. Following this humanitarian crisis, funding by the Bill and Melinda Gates Foundation of the AFRICHOL project, a cholera surveillance network in Africa, and above all the roadmap for cholera elimination by 2030, by the World Health Organization (WHO) and the Global Task Force on Cholera Control (GTFCC), gave new impetus to the fight against cholera, mainly in Africa [2]. Unfortunately, a resurgence in cholera cases has been observed in many countries throughout Africa in 2023, including Malawi, Mozambique, Zimbabwe, Kenya, Ethiopia, Somalia, Democratic Republic of the Congo (DRC), Cameroon, Nigeria, and most recently Sudan [3,4], despite the adoption of Multisectoral Cholera Elimination Plans in many countries guided by the GTFCC.

Climate and other factors that drive cholera transmission are multifaceted and complex. Notably, El Niño, a climate phenomenon characterized by abnormal warming of the Pacific Ocean leading to significant changes in weather conditions worldwide, has been linked to increases in cholera cases [5]. Reduced rainfall due to El Niño can result in water scarcity and insufficient access to clean water sources, forcing vulnerable communities to use contaminated water and reduce proper hygiene practices (handwashing). Meanwhile, heavy rainfall and flooding associated with El Niño can lead to contamination of drinking water sources and disrupt sanitation infrastructures. Both scenarios increase the risk of cholera transmission. In 2017, cholera cases increased by 828% and deaths increased by 133% worldwide compared to 2016 [6]. This increase was due in part to heavy rainfall, which led to sewage overflows and subsequent contamination of drinking water sources. Indeed, the year 2017 followed one of the strongest El Niño events on record from 2015 to 2016. A direct link between the El Niño phenomenon and the cholera epidemics reported worldwide in 2017 has not been clearly established; however, earlier El Niño events have been associated with cholera in Latin America [7]. Previous studies have shown that in Africa, El Niño phenomena are associated with increased rainfall in East Africa and decreased rainfall in Southern Africa, West Africa, and parts of the Sahel [5].

Following the onset of El Niño conditions in July 2023, the latest forecasts indicate a greater than 80% chance of El Niño continuing through March-May of 2024 [8]. Given the links observed between El Niño phenomena and cholera transmission, there is a high risk of increased cholera outbreaks in the near future. Indeed, the WHO has already warned of the risk of an increase in cholera cases worldwide, with over a billion people in 43 countries potentially at risk [9]. Therefore, it is critical to call for urgent, consistent, pragmatic, and coordinated action at all levels to mitigate the impact of these outbreaks on health systems and societies in general. Major donors who prepare for future pandemics should re-evaluate the proportion of funding reserved for pandemics that are already ongoing. Special emphasis should focus on diseases that affect the most vulnerable populations and for which the methods for prevention and control are well understood and available, such as cholera. A meeting to create a “Cholera Elimination Acceleration Fund” should be organized before the end of the year to avoid disastrous epidemics.

Affected and at-risk countries and all supporting agencies (e.g. WHO/GTFCC, Global Action Against Cholera/Veolia Foundation, UNICEF, GAVI, BMGF, International Federation of Red Cross and Red Crescent Societies) should revisit their strategic cholera plans to adapt the geographic targeting of operational response packages considering the challenges posed by El Niño. There is also an urgent need to strengthen country leadership for more intelligent, more effective and better coordinated management of cholera control at all levels. In addition to the “Cholera Elimination Acceleration Fund”, international aid agencies working to combat cholera epidemics should harmonize their approaches to support countries, with each operational strategy adapted according to the updated hotspot classification.
